# Elevated risk thresholds predict endocrine risk-reducing medication use in the Athena screening registry

**DOI:** 10.1038/s41523-021-00306-9

**Published:** 2021-08-03

**Authors:** Yash S. Huilgol, Holly Keane, Yiwey Shieh, Robert A. Hiatt, Jeffrey A. Tice, Lisa Madlensky, Leah Sabacan, Allison Stover Fiscalini, Elad Ziv, Irene Acerbi, Mandy Che, Hoda Anton-Culver, Alexander D. Borowsky, Sharon Hunt, Arash Naeim, Barbara A. Parker, Laura J. van ‘T Veer, Athena Breast Health Network Investigators and Advocate Partners, Laura J. Esserman

**Affiliations:** 1 University of California, San Francisco, San Francisco, CA USA; 2 University of California, Berkeley, Berkeley, CA USA; 3 Peter MacCallum Cancer Centre, Melbourne, Melbourne, VIC Australia; 4 University of California, San Diego, San Diego, CA USA; 5 University of California, Irvine, Irvine, CA USA; 6 University of California, Davis, Sacramento, CA USA; 7 Sanford Health, Sioux Falls, SD USA; 8 University of California, Los Angeles, Los Angeles, CA USA

**Keywords:** Health care, Epidemiology, Cancer screening, Breast cancer, Cancer prevention

## Abstract

Risk-reducing endocrine therapy use, though the benefit is validated, is extremely low. The FDA has approved tamoxifen and raloxifene for a 5-year Breast Cancer Risk Assessment Tool (BCRAT) risk ≥ 1.67%. We examined the threshold at which high-risk women are likely to be using endocrine risk-reducing therapies among Athena Breast Health Network participants from 2011–2018. We identified high-risk women by a 5-year BCRAT risk ≥ 1.67% and those in the top 10% and 2.5% risk thresholds by age. We estimated the odds ratio (OR) of current medication use based on these thresholds using logistic regression. One thousand two hundred and one (1.2%) of 104,223 total participants used medication. Of the 33,082 participants with 5-year BCRAT risk ≥ 1.67%, 772 (2.3%) used medication. Of 2445 in the top 2.5% threshold, 209 (8.6%) used medication. Participants whose 5-year risk exceeded 1.67% were more likely to use medication than those whose risk was below this threshold, OR 3.94 (95% CI = 3.50–4.43). The top 2.5% was most strongly associated with medication usage, OR 9.50 (8.13–11.09) compared to the bottom 97.5%. Women exceeding a 5-year BCRAT ≥ 1.67% had modest medication use. We demonstrate that women in the top 2.5% have higher odds of medication use than those in the bottom 97.5% and compared to a risk of 1.67%. The top 2.5% threshold would more effectively target medication use and is being tested prospectively in a randomized control clinical trial.

## Introduction

Prevention trials have shown unequivocally that endocrine risk-reducing medications (such as selective estrogen receptor modulators and aromatase inhibitors) lower breast cancer risk^1–8^. According to the US Preventative Services Task Force (USPSTF), preventive therapy is recommended for high-risk women, and even younger women^9–12^. However, uptake remains low, for numerous reasons. These reasons include the fear of side effects, resistance to taking a medicine that is used for cancer treatment, objection to taking a medicine at all, the consistent reminder that they are high risk, and failure to appreciate the benefit that they would receive (by both primary care physicians and the woman at risk)^13–15^. Studies have also suggested that patient adherence and use of prevention therapy is correlated to the absolute risk and targeting high-risk groups who would be amenable to risk-reducing therapy^13,16,17^.

A primary care physician’s recommendation often plays a significant role in a patient’s decision-making; risk models were developed to determine risk level and who should consider risk-reducing therapy^18^. The Breast Cancer Risk Assessment Tool (BCRAT) risk model was developed by the National Cancer Institute for use in the National Surgical Adjuvant Breast and Bowel Project (NSABP) P-01 breast cancer prevention trial. As a result, the Food and Drug Administration (FDA) approved the use of selective estrogen receptor modulators (SERMs) tamoxifen and raloxifene in women with a 5-year BCRAT risk of 1.67% or greater, based on the average 5-year breast cancer risk for a 50-year-old white female^7,19,20^. FDA approval of tamoxifen and raloxifene in women with a 5-year BCRAT risk of 1.67% or greater has not led to widespread prescription and uptake of risk-reducing therapy^13,21^.

In the ensuing years, many newer risk models have been developed to identify risk for breast cancer, include BOADICEA, Tyrer-Cuzick, and Breast Cancer Surveillance Consortium (BCSC)^22–24^. The aforementioned models can be modified by genetic, genomic, or polygenic risk, but research findings are still undergoing validation. For example, a number of high and intermediate penetrance susceptibility genes (e.g., ATM, CHEK2, and PALB2) have been found to be associated with breast cancer. The science of single-nucleotide polymorphisms (SNPs) in stratifications of risk are also modifying models that could impact screening and risk assessment^25–27^. To date, some studies have demonstrated that SNPs can improve BCRAT, Tyrer-Cuzick, and BCSC risk models^26,28–33^.

Most risk models are not necessarily calibrated at specific risk thresholds. We identified that the highest risk group—e.g., top 10% risk by age—identifies a group at much higher risk for breast cancer, where women might be motivated to take endocrine risk reduction^34^. Though risk models have improved, it is still not well studied how best to target prevention medications effectively at those who are likely to take them. There is a lack of agreement in published studies on whether risk-reducing therapy use increases when women are at high risk^13^. Since absolute thresholds (such as the FDA approval for tamoxifen and raloxifene) are more likely to overrepresent older women, we hypothesize that age-based thresholds may also include younger women who would benefit from risk-reducing therapy.

The Athena Breast Health Network retrospectively assessed the usage of endocrine risk-reduction therapy by the threshold of risk by age. This program started in 2010, and thus the BCRAT risk model was standard at that time. We sought to determine at what risk threshold therapy is already used by women before standardized active outreach/high-risk counseling, as this may suggest the threshold that primary care providers prescribe, and women use, these therapies. We use a screening registry cohort to compare risk-reducing therapy usage at their time of enrollment in the cohort across three risk thresholds: (1) absolute 5-year BCRAT risk of over 1.67%, (2) top 10% risk by age, and (3) top 2.5% risk by age.

## Results

### Descriptive statistics

Table 1 shows the demographic characteristics of the Athena sample. Of the 104,223 participants, 1210 (1.2%) indicated endocrine risk-reduction use. The highest risk-reducing therapy usage was in risk threshold 3 (top 2.5% risk by age), where 209 of 2445 women (8.6%) used risk-reducing therapy. The lowest risk-reducing therapy usage was in risk threshold 1 (5-year BCRAT risk ≥ 1.67%), where 772 of 33,082 women (2.3%) reported medication use. The average risk was higher in the age-based threshold groups (thresholds 2 and 3). Mean age was slightly older in threshold 1 (61, SD = 7.6), while the age-based thresholds were consistent with the overall sample. The sample was mostly white and non-Hispanic.

**Table 1:** Selected characteristics of the Athena Breast Health Network Screening Registry.

		(1)	(2)	(3)
Characteristics	Total sample (*n* = 104,223)	5-year BCRAT risk ≥ 1.67 (*n* = 33,082)	Top 10% risk by age (*n* = 9,742)	Top 2.5% risk age (*n* = 2,445)
Risk-reduction med (%)				
Medication	1,201 (1.2)	772 (2.3)	410 (4.2)	209 (8.6)
No Medication	103,022 (98.8)	32,310 (97.7)	9,332 (95.8)	2,236 (91.5)
Mean BCRAT 5-year score (SD)	1.5 (1.0)	2.6 (1.2)	3.5 (1.8)	5.3 (2.4)
Mean age (SD)	54 (9.8)	61 (7.6)	55 (9.4)	55 (9.6)
35–44 (%)	21,908 (21.0)	803 (2.4)	1,898 (19.5)	456 (18.7)
45–54 (%)	32,317 (31.0)	5,469 (16.5)	3,168 (32.5)	774 (31.7)
55–64 (%)	30,301 (29.1)	13,857 (41.9)	2,858 (29.3)	741 (30.3)
65–74 (%)	19,697 (18.9)	12,953 (39.1)	1,818 (18.7)	474 (19.4)
Ashkenazi ancestry (%)				
No history	95,912 (92.0)	29,077 (87.9)	8,596 (88.2)	2,146 (87.8)
One parent’s side	1,886 (1.8)	694 (2.1)	199 (2.0)	48 (2.0)
Both parents’ sides	6,425 (6.2)	3,311 (10)	947 (9.7)	251 (10.3)
Race (%)				
White	71,741 (68.8)	26,629 (80.5)	7,701 (79.1)	1,853 (75.8)
Asian	12,580 (12.1)	2,040 (6.2)	921 (9.5)	320 (13.1)
Black or African American	4,357 (4.2)	1,002 (3.0)	187 (1.9)	19 (0.8)
Native Hawaiian/other Pacific Islander	303 (0.3)	62 (0.2)	39 (0.4)	14 (0.6)
Native American or Alaska Native	282 (0.3)	77 (0.2)	31 (0.3)	8 (0.3)
Multiracial or other	4,758 (4.6)	895 (2.7)	357 (3.7)	94 (3.8)
Unknown/decline to answer	10,202 (9.8)	2377 (7.2)	506 (5.2)	137 (5.6)
Ethnicity (%)				
Not hispanic	88,437 (84.9)	30,194 (91.3)	8,809 (90.4)	2,203 (90.1)
Hispanic	11,985 (11.5)	1,747 (5.3)	637 (6.6)	159 (6.5)
Unknown/decline to answer	3,801 (3.7)	1,141 (3.5)	296 (3.0)	83 (3.4)

The dependent and descriptive variables for individuals in the Athena Network registry overall, those in absolute 5-year BCRAT risk ≥ 1.67%, those in the top 10% of 5-year BCRAT risk by age, and those in the top 2.5% of 5-year BCRAT risk by age.

Table 2 describes the distribution of women in various risk strata and their use of risk-reducing medication. The percentage of chemoprevention users in each nonoverlapping risk strata increases from 0.6% among those not meeting the 5-year BCRAT risk to 8.6% among those in the top 2.5% of risk by age. Still, the proportion of no endocrine risk reduction used remained consistent across the strata, with over 90% of women in each nonoverlapping threshold not currently using risk reduction.

**Table 2:** Distribution of high-risk women in comparison to other risk strata.

	5-year BCRAT risk < 1.67%	Risk ≥ 1.67% and below top 10% risk by age	Above top 10% and below top 2.5%	Above top 2.5%
Endocrine risk reduction (%)	429 (0.6)	367 (1.5)	196 (3.2)	209 (8.6)
No endocrine risk reduction (%)	70,712 (99.4)	24,068 (98.5)	6,006 (96.8)	2,236 (91.5)
Total (*n* = 104,223)	71,141	24,435	6,202	2445

The distribution of high-risk women in four nonoverlapping strata in the Athena Network registry: those in absolute BCRAT 5-year risk < 1.67, those above 5-year BCRAT risk ≥ 1.67% and below 10% of risk by age, in top 2.5–10% of risk by age, and those in top 2.5% of risk by age.

The mean risks of each risk threshold stratified by risk-reducing therapy use are provided in Table 3. Notably, among participants indicating risk-reducing therapy use, lifetime score in risk threshold 3 (mean lifetime BCRAT risk = 27.73% or 5-year BCRAT risk = 5.68%) was nearly 1.7 times that of risk threshold 1 (mean lifetime BCRAT risk = 15.90% or 5-year BCRAT risk = 3.52%).

**Table 3:** Mean risk scores for each threshold.

Risk threshold	(1)				(2)					(3)			
	5-year risk ≥ 1.67%		5-year risk < 1.67%		Top 10% by age		Lower 90% by age			Top 2.5% by age		Lower 97.5% by age	
Mean risk	5 year	Life	5 year	Life	5 year	Life	5 year	Life	5 year		Life	5 year	Life
Endocrine risk reduction	3.52%	15.90%	1.11%	7.82%	4.54%	21.71%	1.62%	8.22%	5.68%		27.73%	1.97%	9.74%
No endocrine risk reduction	2.54%	11.66%	1.02%	8.70%	3.31%	18.13%	1.30%	8.70%	5.06%		25.41%	1.41%	9.25%

The mean 5-year and lifetime BCRAT risk for those who use endocrine risk-reducing medication and those that do not within the Athena Network registry for three risk thresholds: absolute risk ≥ 1.67, top 10% of risk by age, and top 2.5% of risk by age. The lifetime BCRAT risk is estimated to age 90. *p* < 0.001 for each threshold (1, 2, 3) using Pearson’s Chi-square test between thresholds and endocrine risk reduction use.

In addition, as described in Supplementary Table 1 and Supplementary Fig. 1, the mean 5-year BCRAT risk varied with each of the different age categories for a given risk by age.

### Regression analysis

The results from three bivariate logistic regression models for risk-reducing therapy adoption and predicted probabilities from marginal effects are presented in Table 4. The models were all significant using the likelihood-ratio chi-squared test to *P-*value < 0.01. Additional regression models, including adjustments by age and continuous 5-year risk, are included in Supplementary Table 2.

**Table 4:** Logistic regression analysis of risk-reducing therapy use in Athena network.

	Regression output				Predicted probabilities			
	Odds ratio	95% CI lower limit	95% CI upper limit	*p* value	Actual % of therapy use	Pred. % of therapy use	95% CI lower limit	95% CI upper limit
(1) 5-year BCRAT ≥ 1.67%	3.94	3.50	4.43	*p* < 0.001	2.38%	2.33%	2.17%	2.50%
(2) Top 10% risk by age	5.20	4.61	5.87	*p* < 0.001	4.20%	4.21%	3.81%	4.61%
(3) Top 2.5% risk by age	9.50	8.13	11.09	*p* < 0.001	9.34%	8.55%	7.44%	9.66%

The logistic regression analyses and predicted probabilities for endocrine risk-reducing medication by three risk thresholds are shown. Predicted probability columns provide diagnostics to test if the model is similar to the actual distribution of therapy used in the data.

Meeting threshold 1 was associated with nearly 4 times higher odds of adopting medication than if a participant did not meet threshold 1 (Odds ratio [OR] = 3.90; 95% confidence interval [95% CI]: 3.50–4.43). The predicted probability of a woman meeting risk threshold 1 reporting medication use was 2.33% (95% CI: 2.17–2.50; actual use in the dataset: 2.38%).

Satisfying threshold 2 was associated with over five times higher odds of reporting medication use than if a participant did not meet threshold 2 (OR = 5.20; 95% CI: 4.61–5.87). The predicted probability of a woman meeting risk threshold 2 reporting risk-reducing therapy was higher, at 4.21% (95% CI: 3.81–4.61; actual use in the dataset: 4.20%).

Satisfying threshold 3 was associated with 9.5 times higher odds of adopting medication than if a participant were not in threshold 3 (OR = 9.50; 95% CI: 8.13–11.09). The predicted probability of an average woman in threshold 3 using risk- reducing therapy was 8.55% (95% CI: 7.44–9.66; actual use in the dataset: 9.34%).

## Discussion

This paper suggests that stratifying risk by age categories was associated with a higher medication use compared with the absolute risk threshold of a 5-year risk ≥ 1.67%. Participants within the top 2.5% of risk by their age (risk threshold 3) had higher odds of risk-reducing therapy use than those below this threshold. Notably, among participants indicating medication use, the lifetime score in risk threshold 3 (mean lifetime score = 27.7%) was 1.7 times that of risk threshold 1 (mean lifetime score = 15.9%).

Our age-based, elevated risk thresholds set for the top 2.5% were generally consistent with the USPSTF recommendation that the benefit of risk-reducing therapy outweighs the risk for women with an absolute 3% 5-year breast cancer risk^12^. However, our top 2.5% by age threshold also captured women ages 35–44 who fell below this USPSTF threshold. Using the absolute USPSTF threshold would have decreased the percentage of women from this younger age category who should be targeted for medication, per our findings. Therefore, age-based risk thresholds may better target prevention among younger women, whose risks may be high for their age categories, but not enough to meet the absolute risk cut-off. These younger women identified through the risk thresholds method are likely to benefit for a longer time course as well, as the pharmacologic benefit of endocrine risk reduction would last beyond the 5 years of active therapy^11^. This risk assessment approach parallels a strategy proposed for cardiovascular disease prevention to move beyond treatment solely on risk models, where risk increases with age^35^.

Our study confirms a strategy to identify those high-risk women who are inclined to use risk-reducing therapy for population health^21^. Prior studies have mainly focused on assessing which components of risk scores are associated with higher risk-reducing therapy use^14^. The risk thresholds approach could be especially helpful to primary care physicians. First, it provides these care providers with a more efficient method of identifying individuals who are most likely to benefit from prevention. Second, previous literature suggests that sharing how a woman’s risk compares to others their own age could improve their understanding of risk^34^. This may help to ameliorate concerns highlighted in studies that have shown that risk assessment could be better communicated to women to increase uptake, with improved cultural humility or low literacy friendly language^15,36,37^.

This study’s findings are also neither specific to the risk model we used nor does it suggest a comparison between modern risk models. Instead, it proposes a method for identifying, targeting, and messaging endocrine risk-reducing therapy toward women identified as part of higher risk by age thresholds. These women are already more likely to take endocrine risk-reducing medication. Our results about identifying elevated risk thresholds by age should be replicable using other modern and current risk models, such as Tyrer-Cuzick, BOADICEA, and Breast Cancer Surveillance Consortium (BCSC)^22–24^.

There remain limitations with our study. It is generally difficult to capture all aspects that influence a person’s decision to adopt or adhere to endocrine risk-reducing therapy, which also involves understanding the risk of known side effects. Athena screening registry data were cross-sectional, prior any Athena high-risk consultations. Therefore, it is impossible to ascertain how much information women received about their risk at a different primary care provider or screening clinic. The calculated risk score for each woman is not necessarily the woman’s risk when she started on endocrine risk-reducing medication, so this may have informed a woman’s decision to take endocrine risk-reducing medication previously. Due to the cross-sectional design, this study also does not assess drug uptake or compliance. Further research needs to be done to assess the efficacy of standardized breast health specialist consultations and to better understand the factors impacting decision-making. A study design with prospective follow-up on the use of endocrine risk-reducing medication among healthy women is needed to confirm our conclusions.

Our analysis is also limited to logistic regression-based solely on risk threshold, which was calculated based on distributions within the Athena screening registry sample. The present sample is limited in its racial diversity (largely white and non-Hispanic), and its geography (participants based in the west and midwestern United States). This study also could be strengthened by predictions with more comprehensive risk factors and the use of a validated large-sample longitudinal, prospective studies, such as those included in the Nurses’ Health Study^34^. Additionally, our retrospective analysis was conducted using 5-year BCRAT risk estimates, because it was standard during the early years of data collection. There also are issues with the BCRAT risk model, highlighted previously, which could be improved upon by more modern risk models and the inclusion of other breast density, genomic, genetic, and polygenic risk factors.

Our study suggests that women in the top 2.5% of risk by age (mean 5-year BCRAT risk of 5.68% or mean lifetime BCRAT risk of 27.73%) are using endocrine risk-reducing medication. The use (8.2%) in this subset, while higher than the national average, is still low. Using age-stratified, percentile-based risk thresholds may be helpful to target endocrine risk-reducing medication and risk-reducing strategies.

To test this finding prospectively and address the limitations of this retrospective analysis, Athena investigators developed the Women Informed to Screen Depending On Measures of risk (WISDOM) Study. The WISDOM Study is a pragmatic, randomized control trial (NCT02620852), recruiting a diversity of participants across the United States representative of the national population. The third principal aim of the study tests if identifying and providing an enriched approach—with direct educational outreach to women in the narrowly defined the top 2.5% of risk by age—increases endocrine risk-reducing medication uptake and acceptability among high-risk women compared to standard risk assessment^38^. The risk thresholds by age will be calculated using the BCSC risk model combined with a polygenic risk score (PRS). To support this aim, a risk-based prevention framework was created. The women in the top 2.5% of risk by age will have direct outreach by a Breast Health Specialist. Investigators developing a standardized presentation of risk and delivering educational materials about risk-reducing strategies through an online risk assessment tool, which will be publicly available once validated^39^. Additionally, risk-reducing lifestyle interventions may be appropriate for an expanded risk group informed by the WISDOM Study. These efforts will hopefully lead to greater use of risk reduction options among populations who would benefit the most.

This and subsequent studies will enable more comprehensive follow-up of women who develop breast cancer who are both above and below these risk thresholds. More women in absolute numbers who develop breast cancer may be below the top 2.5% risk threshold and may be less likely to take up endocrine risk-reducing medication. However, understanding these nuances will ultimately improve our ability to stratify and communicate breast cancer risk, and target risk-reducing options to appropriate high-risk individuals.

## Methods

### Clinical procedures and measurements

The Athena Breast Health Network (Athena) was established in 2009 across the University of California (UC) medical centers (Davis, Irvine, Los Angeles, San Diego, San Francisco), and later added the Sanford Health system in the midwestern United States to recruit a national cohort of women screened with mammography or other technologies^40^. One of Athena’s principal strategic aims is to integrate risk assessment into the breast cancer screening process and identify those at high risk to offer preventive options^25^.

Every woman screened for breast cancer at a participating Athena clinic is required to complete an online intake survey prior to each mammography screening appointment. The survey includes questions regarding a woman’s age, demographic information, family cancer history, and other breast cancer risk factors. In addition, the survey contained a question about the use of risk-reducing medication use, such as SERMs (tamoxifen, raloxifene) and AIs (anastrozole, exemestane, letrozole). Self-reported survey data were taken from each survey and imported into a data warehouse, run by the Salesforce Cloud. The inputs were automatically calculated and stored as 5-year, 10-year, and lifetime BCRAT risk, which was considered standard at the start of the Network.

Once the intake survey was completed, Athena participants received active outreach via telephone if their 5-year BCRAT risk was in the top 10% by age. Breast health specialists (genetic counselors and preventive medicine specialists with expertise in breast cancer risk assessment) provided education on breast cancer risk using a personalized risk assessment framework^34^. The content of the telephone call included a review of the participant’s individual risk and protective factors, clarification of their entered data, and discussion of recommendations for breast cancer prevention and early detection. Breast health specialists explained how the risk model and/or family history criteria were applied to participants; and, engaged in a personalized discussion of early detection and risk reduction options.

The Athena Salesforce platform includes a module for the breast health specialists to document which risk-specific elements were discussed during the phone call, and which (if any) referrals were recommended during the call. Genetic testing was recommended when appropriate based on National Comprehensive Cancer Network (NCCN) National Clinical Practice Guidelines in Oncology^41,42^. The module generated a consult note that was then uploaded to the participant’s electronic medical record. The cost of the BHS consultation was covered by some payers; when insurance did not cover the consultation, the cost was covered by the Athena program.

### Study population

**Fig. 1:**
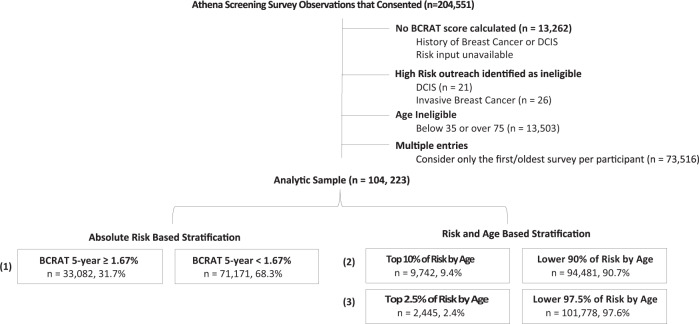
Consort diagram: Athena Breast Health Network breast screening registry. The inclusion and exclusion criteria for the analytic sample in Athena Network registry. (1) refers to threshold 1: FDA approval for tamoxifen and raloxifene is 5-year risk score > 1.67; (2) refers to threshold 2: Use of Top 10% of Risk by Age; (3) refers to threshold 3: Use of Top 2.5% of Risk by Age.

The Athena screening registry was conducted in accordance with protocols approved by the Institutional Review Board (IRB) of the University of California, San Francisco (11–06402). Informed consent was waived by the IRB, given there was no more than minimal risk to human subjects, and involves no procedures for which written consent is normally required outside of the research context. Participants were screened at UC Irvine, UC Los Angeles, UC San Francisco, UC San Diego, and Sanford Health between January 2011 and October 2018. Preliminary data collection was conducted in 2019. A maximum analytic sample of 104,223 women aged 35–74 was retained (Fig. 1) after excluding women with history of ductal carcinoma in situ (DCIS) or invasive breast cancer, for whom no risk was calculated, for those who did not meet age criteria. If a woman completed multiple surveys (e.g., if they had another mammogram in a subsequent year), we only considered the earliest entry.

### Dependent variable

The dependent variable of interest, present medication use, refers to the self-reported use of risk-reducing therapy before screening at an Athena site and Athena high-risk counseling. The therapies included in this category are SERMs (tamoxifen and raloxifene) and aromatase inhibitors (exemestane, letrozole, and anastrozole). This information was self-reported on the Athena intake survey. The use of risk-reducing therapy was cataloged as a binary variable (i.e., “currently using risk-reducing medications” or “not currently using risk-reducing medications”).

### Independent variables

Three risk thresholds were of interest: 5-year BCRAT score ≥ 1.67% (risk threshold 1), top 10% risk by age (risk threshold 2), and top 2.5% risk by age (risk threshold 3).

To calculate whether a particular sample met a particular risk threshold, we used the calculated 5-year risks and lifetime risks (estimated to age 90) for each participant using the BCRAT risk model. Inputs used in the BCRAT model to calculate risk scores are age, race/ethnicity, age of first menstrual period, first live birth, first-degree relatives with breast cancer, and previous breast biopsy^19,43,44^. Both 5-year and lifetime BCRAT risk estimates were provided because different expressions of risk may be salient to patients or their doctors, as documented in the literature^34,45–50^.

For risk threshold 1, we considered if women met the 5-year BCRAT risk score ≥ 1.67%. For risk thresholds 2 and 3, samples were grouped by age at survey completion (categories are ages 35–44, 45–54, 55–64, and 65–74). The risk threshold was calculated by mapping the distribution of risk by age category within the Athena cohort, and determining the top 10% and 2.5% (risk thresholds 2 and 3, respectively) of risk cut-offs by age grouping (see Supplementary Table 1 and Supplementary Fig. 1 for further details).

### Statistical analysis

All statistical analyses were performed using Stata version 16.1^51^. We used each of the three risk thresholds to predict self-reported current risk-reducing therapy use. The unit of analysis is an individual participant. A logistic regression model estimated risk-reducing therapy use based on each of the risk thresholds. Odds ratios (ORs) for all regression coefficient estimates were calculated. In addition to odds ratios, predicted probabilities were also calculated to assess the logistic regression model and provide an estimated probability of risk-reducing therapy use based on the average individual in each risk threshold. Predicted probabilities can be used for diagnostics and validation of the logistic regression model^52^. To control for Type I error, we set our significance level at *p* value < 0.05, with 95% confidence intervals.

### Reporting summary

Further information on research design is available in the Nature Research Reporting Summary linked to this article.

## Supplementary information

Reporting Summary

Supplementary Information

## Data Availability

The data generated and analysed during this study are described in the following data record: 10.6084/m9.figshare.14778675^53^. Relevant data code that support the analysis of data for the study are available in the figshare repository at 10.6084/m9.figshare.14444546.^54,55^ Data analysis was conducted with Stata 16.1.

## Code availability

Code that supports the analysis of data are available from the corresponding author upon reasonable request.
