# Supplementary material for: Elevated risk thresholds predict endocrine risk-reducing medication use in the Athena screening registry
Source: NPJ Breast Cancer. 2021 Aug 3;7:102. doi: 10.1038/s41523-021-00306-9 (PMC8333106; doi:10.1038/s41523-021-00306-9)
Supplement: Supplementary file 1 — Reporting Summary [file 41523_2021_306_MOESM1_ESM.pdf]

# nature research

Corresponding author(s): Esserman, L.J.

Last updated by author(s): Apr 4, 2021

# Reporting Summary

Nature Research wishes to improve the reproducibility of the work that we publish. This form provides structure for consistency and transparency in reporting. For further information on Nature Research policies, see our Editorial Policies and the Editorial Policy Checklist.

## Statistics

For all statistical analyses, confirm that the following items are present in the figure legend, table legend, main text, or Methods section.

| n/a | Confirmed | |
|---|---|---|
| ☐ | ☒ | The exact sample size ($n$) for each experimental group/condition, given as a discrete number and unit of measurement |
| ☐ | ☒ | A statement on whether measurements were taken from distinct samples or whether the same sample was measured repeatedly |
| ☐ | ☒ | The statistical test(s) used AND whether they are one- or two-sided<br>*Only common tests should be described solely by name; describe more complex techniques in the Methods section.* |
| ☐ | ☒ | A description of all covariates tested |
| ☐ | ☒ | A description of any assumptions or corrections, such as tests of normality and adjustment for multiple comparisons |
| ☐ | ☒ | A full description of the statistical parameters including central tendency (e.g. means) or other basic estimates (e.g. regression coefficient) AND variation (e.g. standard deviation) or associated estimates of uncertainty (e.g. confidence intervals) |
| ☐ | ☒ | For null hypothesis testing, the test statistic (e.g. $F$, $t$, $r$) with confidence intervals, effect sizes, degrees of freedom and $P$ value noted<br>*Give P values as exact values whenever suitable.* |
| ☒ | ☐ | For Bayesian analysis, information on the choice of priors and Markov chain Monte Carlo settings |
| ☒ | ☐ | For hierarchical and complex designs, identification of the appropriate level for tests and full reporting of outcomes |
| ☒ | ☐ | Estimates of effect sizes (e.g. Cohen's $d$, Pearson's $r$), indicating how they were calculated |

*Our web collection on statistics for biologists contains articles on many of the points above.*

## Software and code

Policy information about availability of computer code

| | |
|---|---|
| Data collection | Survey data was imported and collected in the Athena Breast Health Network's Salesforce Cloud System. 5-year and lifetime risk was calculated using the National Cancer Institute's Breast Cancer Risk Assessment Tool (BCRAT). |
| Data analysis | Data analysis was conducted with Stata 16.1. Relevant code that supports the analysis of data for this study are available in the figshare repository at the following metadata record: doi.org/10.6084/m9.figshare.14444546. |

For manuscripts utilizing custom algorithms or software that are central to the research but not yet described in published literature, software must be made available to editors and reviewers. We strongly encourage code deposition in a community repository (e.g. GitHub). See the Nature Research guidelines for submitting code & software for further information.

## Data

Policy information about availability of data

All manuscripts must include a data availability statement. This statement should provide the following information, where applicable:

- Accession codes, unique identifiers, or web links for publicly available datasets
- A list of figures that have associated raw data
- A description of any restrictions on data availability

Relevant data and code that support the findings of this study are available in the figshare repository at the following metadata record: doi.org/10.6084/m9.figshare.14444546.

# Field-specific reporting

Please select the one below that is the best fit for your research. If you are not sure, read the appropriate sections before making your selection.

☐ Life sciences ☒ Behavioural & social sciences ☐ Ecological, evolutionary & environmental sciences

For a reference copy of the document with all sections, see nature.com/documents/nr-reporting-summary-flat.pdf

# Behavioural & social sciences study design

All studies must disclose on these points even when the disclosure is negative.

| | |
|---|---|
| Study description | Retrospective, quantitative observational study. |
| Research sample | Women ages 40-74 who screened for the first time at an Athena Breast Health Network site between January 2011-October 2018. |
| Sampling strategy | N = 104, 223 participants were considered as part of the retrospective, observational study. This was sufficiently large to conduct statistical analyses. |
| Data collection | Every woman screened for breast cancer at a participating Athena clinic is required to complete an online intake survey prior to each mammography screening appointment. The survey includes questions regarding a woman's age, demographic information, family cancer history, and other breast cancer risk factors. Self-reported survey data were taken from each survey and imported into a data warehouse, run by the Salesforce Cloud. The inputs were automatically calculated and stored as 5-year, 10-year, and lifetime BCRAT risk, which was considered standard at the start of the Network. |
| Timing | Data was collected for the time frame January 2011-October 2018. Preliminary data extraction was conducted in 2019. |
| Data exclusions | See CONSORT Diagram (Figure 1) |
| Non-participation | Participants wishing not to consent were given the option to do so. Consent could be withdrawn at any point. |
| Randomization | N/A |

# Reporting for specific materials, systems and methods

We require information from authors about some types of materials, experimental systems and methods used in many studies. Here, indicate whether each material, system or method listed is relevant to your study. If you are not sure if a list item applies to your research, read the appropriate section before selecting a response.

## Materials & experimental systems

| n/a | Involved in the study | |
|---|---|---|
| ☒ | ☐ | Antibodies |
| ☒ | ☐ | Eukaryotic cell lines |
| ☒ | ☐ | Palaeontology and archaeology |
| ☒ | ☐ | Animals and other organisms |
| ☐ | ☒ | Human research participants |
| ☐ | ☒ | Clinical data |
| ☒ | ☐ | Dual use research of concern |

## Methods

| n/a | Involved in the study | |
|---|---|---|
| ☒ | ☐ | ChIP-seq |
| ☒ | ☐ | Flow cytometry |
| ☒ | ☐ | MRI-based neuroimaging |

## Human research participants

Policy information about studies involving human research participants

| | |
|---|---|
| Population characteristics | See above. |
| Recruitment | Participants were recruited and consented to participate as part of their screening appointment at an Athena site. The Athena screening registry was conducted in accordance with protocols approved by the Institutional Review Board (IRB) of University of California, San Francisco (11-06402). Any additional informed consent was waived by the IRB, given there was no more than minimal risk to human subjects, and involves no procedures for which written consent is normally required outside of the research context. |
| Ethics oversight | University of California, San Francisco (UCSF) Institutional Review Board (11-06402) |

Note that full information on the approval of the study protocol must also be provided in the manuscript.

## Clinical data

Policy information about clinical studies

All manuscripts should comply with the ICMJE guidelines for publication of clinical research and a completed CONSORT checklist must be included with all submissions.

| | |
|---|---|
| Clinical trial registration | NCT02620852 |
| Study protocol | NCT02620852 |
| Data collection | N/A - Clinical trial (The WISDOM Study) was mentioned in the discussion |
| Outcomes | N/A - Clinical trial (The WISDOM Study) was mentioned in the discussion. |
