# Supplementary material for: Elevated risk thresholds predict endocrine risk-reducing medication use in the Athena screening registry
Source: NPJ Breast Cancer. 2021 Aug 3;7:102. doi: 10.1038/s41523-021-00306-9 (PMC8333106; doi:10.1038/s41523-021-00306-9)
Supplement: Supplementary file 2 — Supplementary Information [file 41523_2021_306_MOESM2_ESM.pdf]

Article

Elevated Risk Thresholds Predict Endocrine Risk Reducing Medication Use: Findings from the Athena Breast Health Network Screening Registry

Huilgol YS, et al.

Supplementary Information:

Supplementary Table 1. 5-Year BCRAT Risk Score Thresholds Corresponding to Age Categories

Description: Provides thresholds used for risk stratification. The FDA recommendation for tamoxifen and raloxifene is a 5-year BCRAT risk score ≥ 1.67%. Those thresholds meeting the FDA recommendation are in **bold**. All risk thresholds shown with 5-year BCRAT score calculations. Risk thresholds used in analysis based on sample in Athena distribution by four categories of age: 35-44, 45-54, 55-64, and 65-74.

Data from the Nurses' Health Study Gail Scores for comparison, courtesy of Beverly Rockhill et al, Channing Laboratory, Harvard Medical School, and published elsewhere.[1]

Supplementary Table 2. Chemoprevention Use and Risk Thresholds, Stratified by Age

Description: Eight logistic regression analyses (1, 3, 5, and 7 are unadjusted) for Endocrine Risk-Reducing Medication for two thresholds are shown, stratified with younger (age < 45) and older women (age ≥ 45).

Supplementary Table 3. Endocrine Risk Reduction Use at First Screening by Athena Site

Description: Provides the distribution of chemoprevention use in the top 2.5% of risk by age and overall by Athena screening site.

Supplementary Figure 1. Distribution of Risk in Athena Breast Health Network and Thresholds

Description: Presents the distribution of natural logarithm transformed 5-year BCRAT risk in the Athena Breast Health Network at first chemoprevention use and cut offs for different age categories for the top 2.5% of risk by age. FDA allows endocrine risk reduction medication for 5-year BCRAT Score ≥ 1.67% (dotted vertical line 1). Risk thresholds for Top 2.5% used in analysis based on sample in Athena distribution by four categories of age, with threshold values from Supplemental Figure A: 35-44 (Vertical Line 2); 45-54 (Vertical Line 3); 55-64 (Vertical Line 4); 65-74 (Vertical Line 5)

Supplementary Note 1. Member List for Athena Breast Health Network Investigators and Advocate Partners (in Alphabetical Order)

Supplementary Table 1. 5-Year BCRAT Risk Score Thresholds Corresponding to Age Categories

**Risk Threshold**	**FDA**	**Top 10% by age**		**Top 2.5% by age**
**Cohort**	N/A	*Athena*	*Nurses' Health Study*	*Athena*
35-44	**1.67%**	1.22%	Unavailable	**1.89%**
45-54	**1.67%**	**1.96%**	45-49: 1.5%	**2.98%**
			**50-54: 1.7%**	
55-64	**1.67%**	**2.93%**	**55-59: 2.2%**	**4.63%**
			**60-64: 2.7%**	
65-74	**1.67%**	**3.45%**	**65+: 3.2%**	**5.49%**

Supplementary Table 2. Chemoprevention Use and Risk Thresholds, Stratified by Age

	(1)	(2)	(3)	(4)	(5)	(6)	(7)	(8)
	**Absolute Risk Threshold**		**Age Based Threshold**		**Absolute Risk Threshold**		**Age Based Threshold**	
	**Age < 45**				**Age ≥ 45**			
**5-yr BCRAT Risk ≥ 1.67 (Threshold 1)**	10.35***	3.78***			3.40***	1.91***		
	(2.15)	(1.26)			(0.22)	(0.145)		
**Age**		0.81***		0.82***		1.01		1.03***
		(0.03)		(0.03)		(0.004)		(0.004)
**5-yr BCRAT Risk**		1.82***		1.82***		1.36***		1.27***
		(0.20)		(0.22)		(0.02)		(0.03)
**Top 2.5% by Age (Threshold 3)**			14.46***	3.99***			8.90***	2.79***
			(3.23)	(1.61)			(0.75)	(0.42)
**Observations**	21,908	21,908	21,908	21,908	82,315	82,315	82,315	82,315

Standard errors of the odds ratio are provided in parentheses below the odds ratio.
*** p<0.01, ** p<0.05, * p<0.1

Supplementary Table 3. Endocrine Risk Reduction Use at First Screening by Athena Site

Athena Site	Chemoprevention (% of Overall)	Site Total Overall	Chemoprevention (% of Top 2.5%)	Site Total Top 2.5% by age
Sanford Health	141 (0.6%)	25,389	12 (2.4%)	501
UC Irvine	68 (0.6%)	12,187	4 (1.9%)	215
UC Los Angeles	851 (2.0%)	42,801	160 (13.6%)	1,181
UC San Diego	79 (0.6%)	12,019	23 (9.1%)	253
UC San Francisco	62 (0.5%)	11,827	10 (3.4%)	295
**Total Across Sites**	**1,201 (1.2%)**	**104,223**	**209 (8.6%)**	**2,445**

Supplementary Figure 1. Distribution of Risk in Athena Breast Health Network and Thresholds

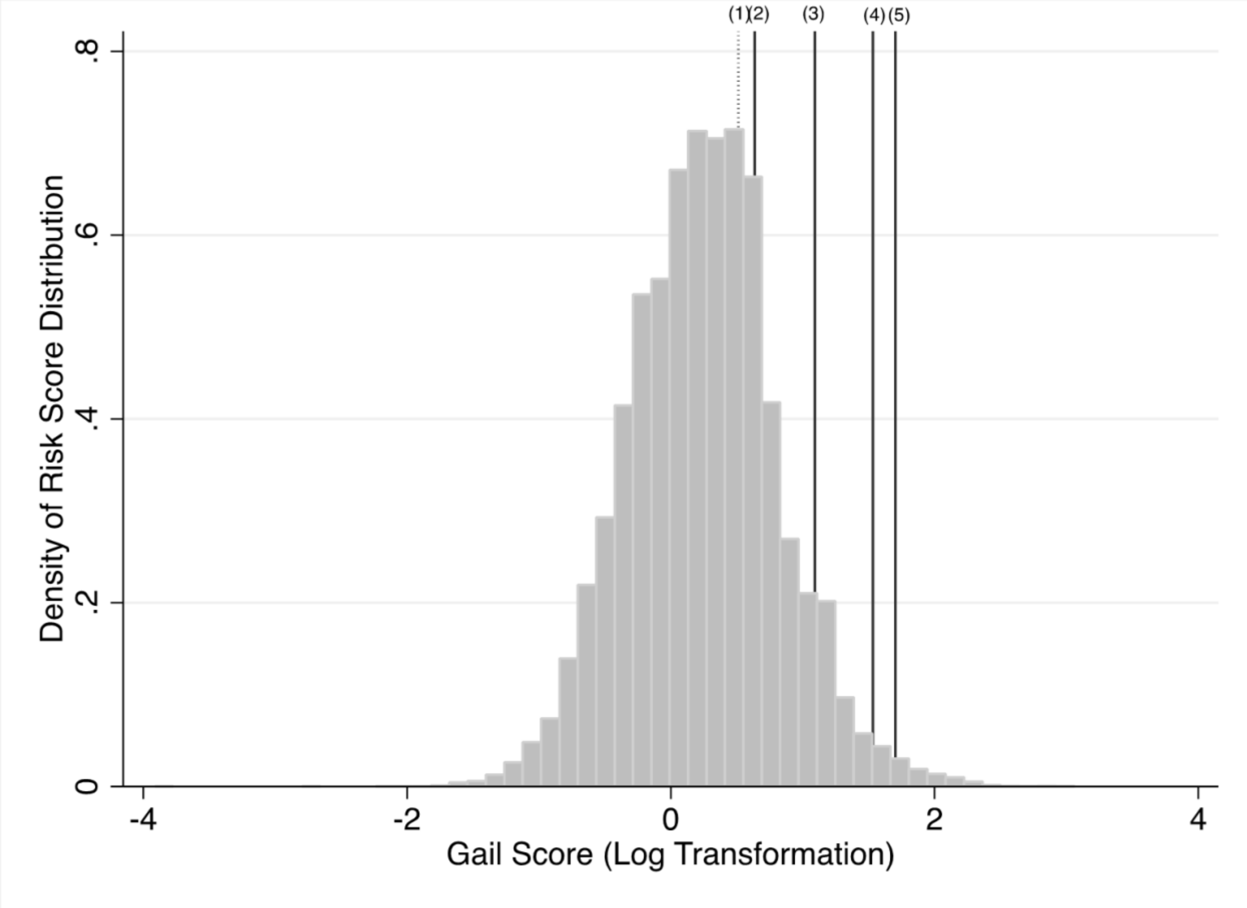

Supplementary Note 1. Member List for Athena Breast Health Network Investigators and Advocate Partners (in Alphabetical Order)

Consortium List: Nancy Anderson[7], Hoda Anton-Culver[5], Alexander Borowsky[6], Susie Brain[1], Beth Crawford[1], Laura Esserman[1], Joshua Fenton[8], Melanie Fiorella[4], Allison Stover Fiscalini[1], Deborah Goodman[5], Heather Harvey[7], Diane Heditsian[1], Robert A. Hiatt[1], Michael Hogarth[6], Sharon Hunt[7], Celia Kaplan[1], Andrea Kaster[7], Kathryn M. Larsen[5], Vivian Lee[1], Lisa Madlensky[4], Arash Naeim[8], Hannah L. Park[5], Barbara A. Parker[4], Antonia Petruse[8], Larissa Risty[7], Yiwey Shieh[1], Jeffrey Tice[1], Barry Tong[1], Laura van 'T Veer[1], Neil Wenger[8], Elad Ziv[1]

Affiliations:
1. University of California, San Francisco, San Francisco, CA, USA
2. University of California, Berkeley, Berkeley, CA, USA
3. Peter MacCallum Cancer Centre, Melbourne, Victoria, Australia
4. University of California, San Diego, San Diego, CA, USA
5. University of California, Irvine, Irvine, CA, USA
6. University of California, Davis, Sacramento, CA, USA
7. Sanford Health, Sioux Falls, SD, USA
8. University of California, Los Angeles, Los Angeles, CA USA

REFERENCES

1. Ozanne, E. M., Klemp, J. R. & Esserman, L. J. Breast Cancer Risk Assessment and Prevention: A Framework for Shared Decision-Making Consultations. *Breast J.* **12**, 103–113 (2006).
